# Comparison of the Acute Effects of Two Neoprene Knee Sleeves on Balance and Pain in Knee Osteoarthritis: A Randomized, Single-Blinded, Prospective Study

**DOI:** 10.5152/ArchRheumatol.2025.10982

**Published:** 2025-06-23

**Authors:** Burcu Ayık, Nurcan Kağan, Fulya Bakılan, Onur Armağan, Cengiz Bal

**Affiliations:** 1 Department of Physical Medicine and Rehabilitation, Eskişehir Osmangazi University Faculty of Medicine, Eski̇şehi̇r, Türkiye; 2 Department of Biostatistics, Eskişehir Osmangazi University Faculty of Medicine, Eski̇şehi̇r, Türkiye

**Keywords:** Balance, knee sleeve, osteoarthritis, posturography

## Abstract

**Background/Aims:**

The aim of this study was to compare the acute effects of 2 different elastic neoprene knee sleeves, 1 equipped with 4 metal supports, on balance and pain in patients with knee osteoarthritis (KOA).

**Materials and Methods:**

A total of 60 patients (50 females, 10 males; age mean = 61.13 ± 8.6 years) diagnosed with KOA were randomly divided into 2 groups. Group 1(n = 30): wearing an elastic neoprene knee sleeve, and group 2 (n = 30): wearing a neoprene knee sleeve with 4 metal supports. The Berg Balance Scale (BBS), Timed Up and Go, Functional Reach Test, and Fall Index, calculated using posturography (Tetrax®), and the Visual Analogue Scale were employed for the assessment of balance and pain. Clinical assessments were performed before and after wearing the knee sleeves. The sample size was determined by power analysis using balance data (*α *= 0.05, power = 0.99).

**Results:**

Both groups exhibited statistically significant improvement in intragroup comparisons of all assessment parameters (*P* < .05). However, the changes in BBS score after wearing the knee sleeve were better in favor of group 2, approaching statistical significance (*P* = .056).

**Conclusion:**

Both types of neoprene knee sleeves provided immediate benefits in balance and pain among patients with KOA. While the neoprene knee sleeve with 4 metal supports showed slightly greater balance improvement, the difference was not statistically significant. Further long-term studies with larger samples are needed to clarify the effects of different knee sleeves on balance and pain in patients with KOA.

## Introduction

**Main Points**

- Both types of neoprene knee sleeves provided immediate improvement in balance and pain in patients with knee osteoarthritis (KOA).
- The knee sleeve with four metal supports showed slightly greater improvement in balance compared to the elastic knee sleeve.
- Knee sleeves may serve as a useful non-pharmacological intervention for managing balance and pain in KOA.

Knee osteoarthritis (KOA) is a prevalent rheumatologic and degenerative joint disease. Knee osteoarthritis, which increases in prevalence with advancing age, causes progressive loss of physical function and daily life activity limitation, leading to a reduction in quality of life.^1,2^

Balance, crucial for daily life activities, is negatively impacted by knee osteoarthritis because, in addition to changes in the articular surface, alterations in the meniscus, ligaments, tendons, and periarticular muscle tissues are also observed in knee osteoarthritis.^3^ Patients with KOA exhibit muscle weakness, impaired proprioception, and a reduction in mechanosensory receptors in the ligaments of the affected joints.^4,5^ Consequently, the ability to control the body’s center of balance and maintain postural stability is compromised.^6-8^ Poor balance control is more likely to experience falls and difficulties in mobility.^9^ In addition, a decline in cognitive function has been observed in individuals with chronic pain conditions such as knee osteoarthritis.^10^ It is postulated that this may contribute to an impairment of balance.

The goal of KOA management is to alleviate pain, rectify mechanical malalignment, and manage symptoms associated with the knee joint. Treatment for this purpose includes conservative and surgical therapies. Surgery should be considered a last-resort treatment in advanced cases of KOA.^11^ Conservative treatment management strategies encompass weight reduction, physiotherapy, structured exercise programs, orthotic support, and medication-based therapies.^12^ Orthotic devices, including knee braces, wedged insoles, and elastic knee sleeves, are employed to manage symptoms and prevent disease progression.^13,14^ The results of clinical studies indicate that knee sleeves can effectively reduce pain,^15^ provide biomechanical support by reducing the angle of adduction,^16^ and improve functional ability,^15,17,18^ proprioception^4,19^ and postural control^20^ in patients with knee osteoarthritis. Although it has been proven that balance is adversely affected as the severity of knee osteoarthritis increases,^21^ only 1 study investigating the effects of elastic knee sleeves on balance has been reported in the literature to the authors’ knowledge. According to the aforementioned study, Chuang et al^20^ reported that an elastic neoprene knee sleeve improved static and dynamic balance in KOA. However, knee sleeves offer a lesser degree of mechanical support than braces, due to the composition of their elastic materials.^16^ Therefore, in this study, it was hypothesized that a neoprene elastic knee sleeve with metal supports would have a greater acute effect on balance and pain than a neoprene elastic sleeve. A review of the existing literature indicates a lack of studies examining the comparative acute efficacy of different knee sleeve types on balance and pain in KOA.

The aim of this study was to compare the acute effects of 2 different elastic neoprene knee sleeves, 1 equipped with 4 metal supports, on balance and pain in KOA.

## Materials and Methods

This study was conducted as a randomized, single-blind, prospective study. Participants were selected from patients who registered at the Eskişehir Osmangazi University Physical Therapy and Rehabilitation Outpatient Clinic between December 2022 and October 2023. Ethical approval was obtained from the Eskişehir Osmangazi University Clinical Research Ethics Committee (approval number: E-80558721-050.99-413730 / 10.11.2022-24). The patients were informed about the study and written consent was obtained from the patients. The research was carried out according to the Declaration of Helsinki. The study was registered at ClinicalTrials.gov with registration number NCT06813716.

### Patients

This study was conducted on a total of 60 patients between the ages of 45 and 75 who were diagnosed with KOA according to the American College of Rheumatology criteria.

Patients with unilateral or bilateral KOA, Kellgren-Lawrence grade 2 (mild) or 3 (moderate), knee muscle strength of 4 or higher, and those who voluntarily participated and signed informed consent were included. Both knees of patients with bilateral knee osteoarthritis had the same Kellgren-Lawrence scale. Patients with arthroscopy of the knee joint within the last 1 year, knee and/or hip replacement, limitation of joint range of motion, history of inflammatory rheumatic disease, vestibular and cerebellar disease, visual loss of less than 2/10 in both eyes, neurological diseases (e.g., Parkinson’s disease, stroke, ataxia, dementia, multiple sclerosis, peripheral neuropathy) were excluded.

### Sample Size

The sample size was determined according to power analysis. The statistical program MINITAB 16.0 (Minitab Inc., State College, Pennsylvania, USA) was used for power analysis. Since one of the main hypotheses of the study was to compare 2 independent groups according to the Spadi index (quantitative variable), the t-test was considered in the sample size calculation. Based on the TUG score parameter—with an expected mean of 13.97 (SD 2.25) in the first group and an expected mean of 11.65 (SD 1.58) in the second group—it was determined that 23 subjects should be enrolled for each group to have 99% power at a 5% type 1 error level, based on the results of the Munshi et al’s^22^ study. However, in order to increase the power of the study, the number of participants included in the study was increased to 30 in each group.

### Randomization

Patients were randomly assigned to 2 groups using the sealed envelope method, which is a simple randomization method. Each patient selected an envelope labeled either “elastic neoprene knee sleeve” or “neoprene knee sleeve with 4 metal supports.” The assessing physician’s assistant opened the selected envelope and prepared the patients for the assessment.

### Blinding

The process of putting on and taking off appropriate knee sleeves was done with the help of an assistant other than the assessing physician. As the knee sleeves were not visible under the clothes, the assessments were carried out by a blinded physician who was unaware of the existence of the knee sleeve or which type of knee sleeve it was.

### Knee Sleeves

**Figure 1.**
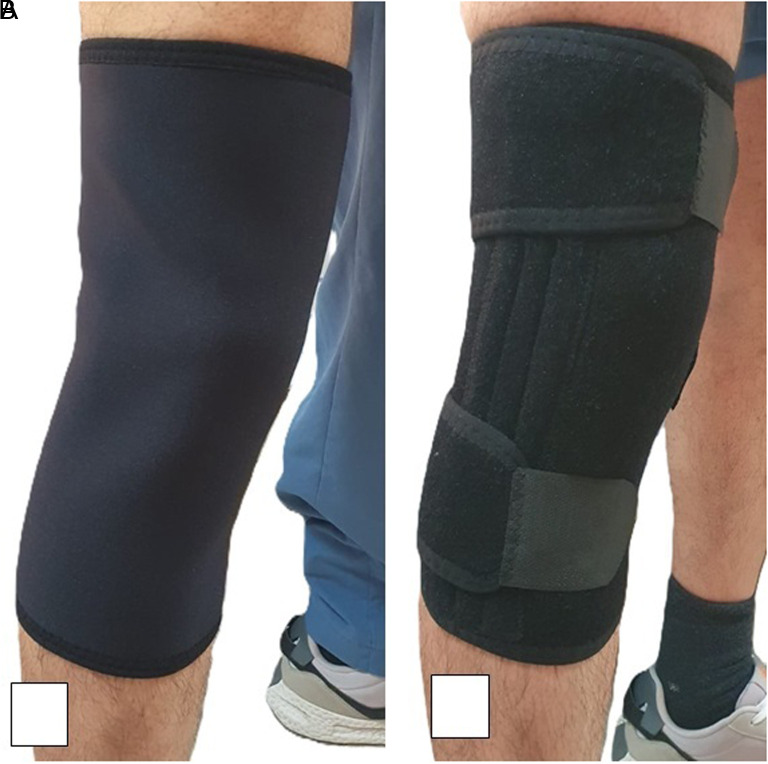
A: Neoprene elastic knee sleeve; B: Neoprene knee sleeve with four metal supports.

Neoprene elastic knee sleeve without patellar opening (ORSA®, model N-31 -Türkiye/Kiwa certified ISO13485) and neoprene knee sleeve with 4 metal supports; 2 supporting bars on each medial and lateral side without patellar opening (ORSA®, model N-31S –Türkiye/Kiwa certified ISO13486) were used in the study (Figure 1).

According to the manufacturer, 5 different sizes of knee sleeves of different sizes were used. Knee circumference measurement: 30 to 33 cm for size S; 33 to 36 cm for size M; 36 to 39 cm for size L; 39 to 42 cm for size XL; 42 to 45 cm for size XXL. The smaller size was selected when the knee size fell between 2 options.

### Clinical Assessments

Static and dynamic evaluations were performed immediately after the patients were fitted with the knee sleeve. A resting period of 10 minutes was incorporated between static and dynamic assessments, followed by an additional 10-minute resting interval between trials with and without knee sleeves. Participants were asked to perform tests (BBS, TUG, and FRT) 3 times; no interventions were performed between the measurements that could have influenced the outcomes. Additionally, no changes occurred in physical or environmental factors such as fatigue, room temperature, lighting, or psychological conditions that might have affected the assessments. The final score was the average of 3 times.

### Berg Balance Scale

It consists of 14 different areas that assess the maintenance of a static position during changes in the orientation of the body’s center of gravity in the categories of sitting, standing, and posture change. Each area is scored between 0-4 and the total score is between 0-56. Fifty-six points indicate perfect balance.^23^

### Timed Up and Go test

The patient was instructed to stand up from the chair, walk 3 meters forward, turn 180°, return to the chair, and sit down. While performing this test, the time between the time the patient stood up from the chair and the time the patient sat down was recorded in seconds with a stopwatch. This test, which evaluates balance function, is reported to have a threshold value above 14 seconds that predicts falls with high sensitivity and specificity.^24^

### Functional Reach Test

The patient is asked to stand with the arm flexed at 90° and the fist closed, not touching the wall but standing next to it. The assessor marks the head of the third metacarpal on the wall and asks the patient to reach forward as far as possible without taking a step. The assessor again marks the head of the third metacarpal on the wall and the difference between the start and end is measured with a tape measure. Three attempts were made and the average of these attempts was taken. A distance of 15 cm or less signifies a significantly increased risk of falling, while 15 to 25 cm indicates a moderate risk.^25^

### Fall Index Calculation with Tetrax

Each participant was provided with a minimum of 5 minutes for acclimation to the balance platform before testing commenced.

**Figure 2.**
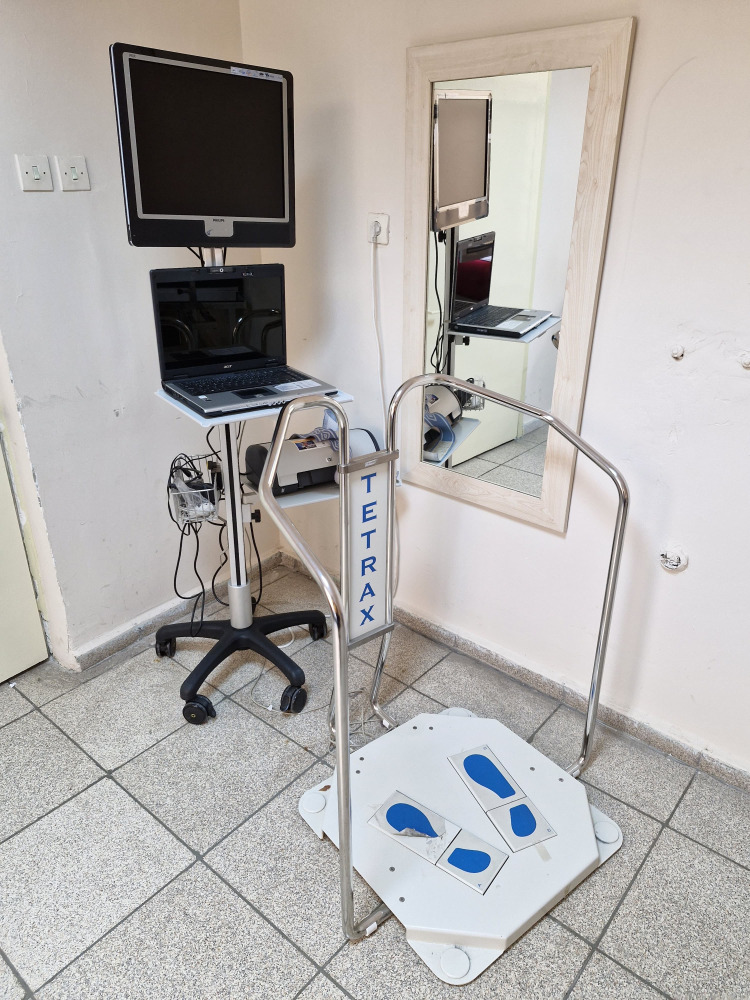
Posturography device.

The fall index was calculated using the software program of Tetrax® (Sunlight Medical Ltd., Ramat Gan, Israel), a Tetra-ataxiometric posturography system, which is the device used to evaluate static balance. This posturography assesses postural sway by measuring weight shifts on 4 separate force plates under the left and right forefoot and hindfoot. The patients were instructed to stand on the platform without shoes and the test was performed in 8 different positions: eyes open, eyes closed, eyes open on a pillow, eyes closed on a pillow, head turned to the right and left with eyes closed, head flexed 30 degrees backward and 30 degrees forward with eyes closed. The test was measured for 32 seconds for each position. The standard eye-opening position serves as a reference for comparison. The risk of falling increases as the fall index increases^26^ (Figure 2).

### Visual Analogue Scale

Patients rated their pain on a scale from 0 to 10, where 0 meant no pain and 10 represented the most intense pain they had ever experienced. In patients with bilateral KOA, the VAS score of the most painful knee was evaluated.

### Statistical Analysis

Data analysis was performed using IBM SPSS for Windows 21 (IBM SPSS Corp.; Armonk, NY, USA). The Shapiro–Wilk test assessed the suitability of variables for normal distribution. Groups were compared using both parametric and nonparametric tests. Groups were compared using Student’s *t*-test or the Mann–Whitney *U *test, depending on the distribution. Paired samples *t*-test and Wilcoxon *t*-test were used to compare data before and after knee sleeve use. The repeated measures analysis of variance (RM-ANOVA) test was used in the analysis of repeated 3 measurements for the performance tests BBS, TUG, and FRT. The intraexaminer reliability of BBS, TUG, and FRT measurements and the agreement between the measurements were evaluated by calculating the intraclass correlation coefficient (ICC). A linear mixed model was used to compare mean differences between groups. Chi-square tests were used to analyze the crosstabs. When summarizing data, the number (%) statistic was used for qualitative data and the mean ± SD or median (25%-75%) statistic was used for quantitative data. A *P*-value of <.05 was considered significant.

## Results

**Figure 3.**
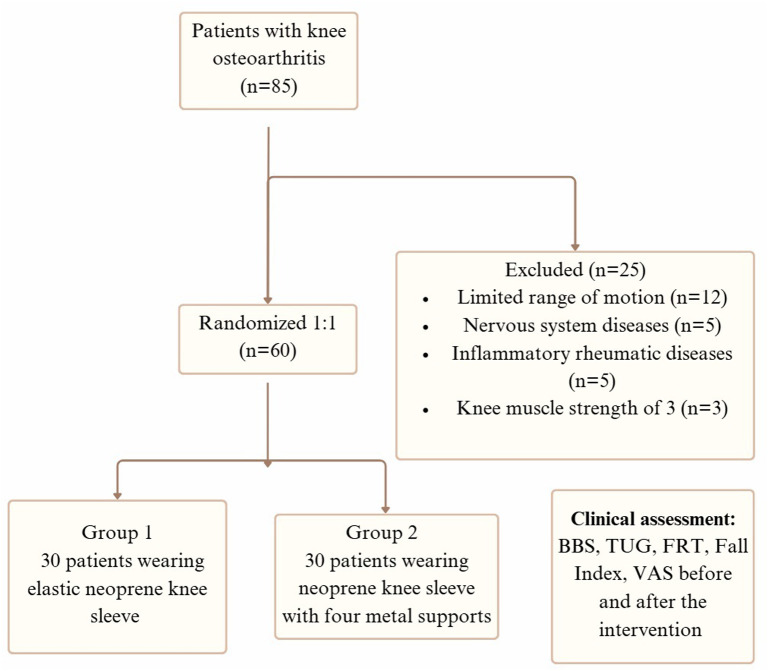
Study flowchart.

Initially, 85 patients diagnosed with knee osteoarthritis were examined for eligibility. Of these, 25 were excluded based on the study’s inclusion and exclusion criteria. The remaining 60 participants were randomized into 2 groups: Group 1 (n = 30), who wore an elastic neoprene knee sleeve, and Group 2 (n = 30), who wore a neoprene knee sleeve with 4 metal supports (Figure 3).

There were no statistically significant differences between the groups regarding age, gender, body mass index, symptom duration, dominance side, affected side, and Kellgren–Lawrence scale (*P* > .05) (Table 1).

In general, the ICC values indicating intraexaminer reliability of repeated measurements were high. For group 1, the ICC was 0.993 (95% CI; 0.988, 0.997) between the BBS (Berg Balance Scale) scores measured 3 times before wearing the knee sleeve, the ICC of the BSS values after wearing the knee sleeve was 0.993 (95% CI; 0.987, 0.996). The ICC between the TUG scores measured 3 times before and 3 times after wearing the knee sleeve was 1 (95%CI; 1, 1), the ICC between the FRT scores measured 3 times before and 3 times after wearing the knee sleeve was 0.999 (95% CI; 0.999, 1). For group 2, the ICC was 0.993 (95% CI; 0.986, 0.996) between the BSS scores measured 3 times before wearing the knee sleeve, the ICC of the BSS scores after wearing the knee sleeve was 0.990 (95% CI; 0.982, 0.995). The ICC between the TUG scores measured 3 times before and 3 times after wearing the knee sleeve was 1 (95% CI; 1, 1), the ICC between the FRT scores measured 3 times before and 3 times after wearing the knee sleeve was 0.999 (95% CI; 0.999, 1).

According to the Shapiro–Wilk test, only VAS values were normally distributed both before and after knee sleeve use (*P* > .05).

Intra-group analysis revealed a significant improvement in BBS, TUG, and FRT scores following sleeve wearing in both groups (*P* < .001 for all).

When comparing between groups, although Group 2 demonstrated slightly higher BBS scores at all post-sleeve assessments compared to Group 1, the differences were not statistically significant (*P* = .082 at baseline and *P* = .056 after sleeve wearing).

Likewise, the TUG and FRT scores did not differ significantly between the groups at any measurement point (*P* > .05 for all comparisons) (Table 2).

The fall index significantly decreased in both groups after sleeve wearing (*P* < .001 for each group). However, no statistically significant difference was observed between the groups regarding fall index values either before (*P* = .160) or after sleeve wearing (*P* = .515) (Table 3).

Visual Analogue Scale scores also showed a significant reduction within each group following sleeve wearing (*P* = .002 for Group 1; *P* = .005 for Group 2). Nonetheless, the comparison between groups demonstrated no significant differences in VAS scores at either baseline (*P* = .145) or after sleeve wearing (*P* = .180) (Table 3).

## Discussion

In this study, both participant groups exhibited significant enhancements in all parameters of balance assessment and experienced pain reduction after using knee sleeves. However, the change in BSS scores after knee sleeve use improved slightly more in the elastic neoprene knee sleeve with 4 metal supports group than in the elastic neoprene knee sleeve group, approaching but not reaching statistical significance.

The balance, which consists of static and dynamic components, is related to the locomotor response given as a result of the processing of sensory inputs from the somatosensory (proprioception), visual, and vestibular systems.^27,28^ Balance is assessed by various assessment methods. The BBS and TUG tests are internationally recognized, internationally accepted, cost-effective, and simple to administer. They are reliable and valid tests for the functional assessment of dynamic balance.^23,29^ Moreover, when TUG and FRT were assessed collectively, the balance assessment demonstrated a correlation with BBS outcomes.^30^ The results of a study conducted on 200 elderly individuals indicate that the TUG and FRT are valid and reliable tools for assessing potential fall risk in this age group.^31^ Therefore, in this study, the dynamic balance was evaluated clinically with BBS, TUG, and FRT. Static and dynamic balance is assessed objectively with various devices. For example, Chuang et al^20^ observed enhanced balance abilities, assessed by an instrument, in both static and dynamic balance in the knee sleeve group when compared to the control group in KOA. In the present study, the fall index was calculated using Tetrax posturography to evaluate static balance.

The balance function in patients with KOA is influenced by age, body mass index (BMI), disease severity, muscle strength, pain, and knee alignment. The musculoskeletal system, various sensory inputs, and the central nervous system responsible for sensorimotor integration are all impacted by aging, potentially leading to a decline in balance with increasing age. Furthermore, the prevalence and severity of disease tend to increase with age.^32^ Kim et al^33^ utilized a variety of clinical assessments, including the BBS, the TUG, and the Tetrax® posturography test to evaluate the relationship between balance and KOA severity. The clinical assessment tools and the Tetrax® test both demonstrated significant differences between the mild, moderate-to-severe, and control groups.^33^ In addition to quadriceps muscle strength being the primary muscle strength for postural balance, a positive correlation has been identified between muscle strength and balance in knee osteoarthritis.^34,35^ In this study, there were no differences in age or disease severity between the patients in the 2 groups, and muscle strength was similar.

A negative correlation was reported between BMI and balance ability. This is because in patients with a high BMI, there is an increase in cartilage degeneration, which in turn leads to an increase in movement limitation and pain, as well as a decrease in muscle strength.^36,37^ This study revealed no notable difference between the 2 groups, despite the mean BMI being above 30, indicative of obesity, which could impair balance function in both groups.^38^

The results of this study indicate that both knee sleeve groups exhibited a markedly beneficial acute effect on pain. A knee sleeve can provide both warmth and consistent compression, which enhances proprioception in the knee joint.^39,40^ Moreover, the knee sleeve reduces the force exerted on the medial compartment of the knee by diminishing the knee adduction moment and impulse. These factors may contribute to the reduction of pain by knee sleeve. Furthermore, there is a decrease in pressure on Hoffa’s fat pad, which is commonly inflamed in knee osteoarthritis, due to compression of the extensor compartment, resulting in decreased pain.^41^ Barrett et al^4^ found a moderate reduction in pain and an increased sense of joint support when using elastic knee braces in their study. Similarly, Bryk et al^15^ investigated the immediate impact of wearing an elastic knee sleeve on pain levels and functionality, observing significant improvements in the VAS score, TUG, and 8-meter walk test.

One of the limitations of the study is that the effects of long-term elastic knee sleeve use on balance and pain were not evaluated. Another limitation is that biomechanical evaluations to assess knee malalignment, which may affect balance, were not performed. Given the absence of functional tests for dynamic balance prediction of falls during active movement, a dynamic performance device is necessary in future studies.

The results of this study indicated that the BBS scores improved slightly more in the group using neoprene knee sleeves with 4 metal supports, but this improvement remained close to statistical significance. Future clinical investigations with expanded sample sizes are required to elucidate this observed difference with greater statistical precision. In addition, the findings suggest that the use of both elastic knee sleeves may confer benefits with respect to static and dynamic balance, falls, and pain. Further research is required to evaluate the effects of different knee sleeves on balance and pain in patients with KOA, utilizing longer study periods and more diverse objective assessment tools.

## Figures and Tables

**Table 1.** Demographics Feature of Groups

	Group 1 (n:30) Mean ± SD	Group 2 (n:30) Mean ± SD	*P*
Age (years)	61.17±9.11	61.10±8.21	.976*
BMI (kg/m²)	30.29±3.40	31.19 ±4.51	.389*
Gender (n: females/males)	25/5	25/5	1.000#
Dominance side (n: right/left)	28/2	27/3	1.000#
Affected side (n: right/left/ bilateral)	9/10/11	13/9/8	.534#
Kellgren-Lawrence scale (n: grade 2/3)	18/12	20/10	.592#

BMI, Body mass index.

*Analyzed by independent samples *t*-test.

#Analyzed by the chi-square tests.

**Table 2.** Intra- and Inter-Group Comparisons of Repeated Assessment Parameters

	Measurement	Group 1 (n:30) Mean ± SD	Group 2 (n:30) Mean ± SD	Group1-Group2 Mean Difference (SEM)	Pairwise Comparisons ***P***
BBS-before sleeve	1st	47.83 ± 6.09	50.57 ± 5.47	−2.72(1.43)	.082
	2nd	48.57 ± 5.82	51.30 ± 5.20		
	3rd	49.33 ± 5.61	52.03 ± 4.97		
BBS-after sleeve	1st	49.33 ± 5.68	52.13 ± 5.36	−2.63 (1.35)	.056
	2nd	50.03 ± 5.42	52.67 ± 5.03		
	3rd	50.73 ± 5.18	53.2 ± 4.73		
	*P*ª	**<.001**	**<.001**		
TUG-before sleeve	1st	10.84 ± 3.84	9.77 ± 3.05	1.07 (0.89)	.237
	2nd	10.9 ± 3.86	9.83 ± 3.05		
	3rd	10.96 ± 3.87	9.90 ± 3.06		
TUG-after sleeve	1st	10.68 ± 3.87	9.70 ± 3.02	0.98 (0.90)	.280
	2nd	10.74 ± 3.87	9.76 ± 3.02		
	3rd	10.80 ± 3.88	9.82 ± 3.03		
	*P*ª	**<.001**	**<.001**		
FRT-before sleeve	1st	21.43 ± 6.40	23.40 ± 6.21	−1.91 (1.63)	.247
	2nd	21.50 ± 6.41	23.41 ± 6.21		
	3rd	21.56 ± 6.44	23.48 ± 6.26		
FRT-after sleeve	1st	22.04 ± 6.41	23.53 ± 6.16	−1.49 (1.62)	.366
	2nd	22.11 ± 6.42	23.59 ± 6.21		
	3rd	22.17 ± 6.44	23.66 ± 6.26		
	*P*ª	**<.001**	**.004**		

Analyzed by independent the repeated measures analysis of variance (RM-ANOVA).

BBS, Berg balance scale; FRT, functional reach test; TUG, timed up and go test.

*P*ª: indicates a significant difference between before and after intervention within the group.

**Table 3.** Intra and Inter-Group Comparisons of Assessment Parameters

		Group 1 (n:30) Mean ± SD Median (25%-75%)	Group 2 (n:30) Mean ± SD Median (25%-75%)	***P***
Fall index	Before	53.03 ± 33.07 56 (24-88)	40.97 ± 28.23 37 (18-52)	.160**
	After	37.0 ± 27.17 34.5 (15.5-56)	31.97 ± 24.74 22 (14-47)	.515**
	*P*ª	**<.001** ^†^	**<.001** ^†^	
	Group1-Group2 Mean difference (SEM)	2.62 (6.62)		.693***
VAS	Before	5.53±2.01	4.46±2.50	.145*
	After	5.0±1.94	4.23±2.40	.180*
	*P*ª	**.002** ^‡^	**.005** ^‡^	
	Group1-Group2 Mean difference (SEM)	0.79 (0.56)		.167***

SEM, Standard error of measurement; VAS, Visual analogue scale.

*Analyzed by independent samples *t*-test.

**Analyzed by Mann–Whitney *U* test.

***Analyzed by the linear mixed model.

^†^Analyzed by the Wilcoxon *t*-test.

^‡^Analyzed by the paired samples *t*-test.

Pª: indicates a significant difference between before and after intervention within the group.

## Data Availability

The data that support the findings of this study are available on request from the corresponding author.
